# How effective are traditional methods of compositional analysis in providing an accurate material balance for a range of softwood derived residues?

**DOI:** 10.1186/1754-6834-6-90

**Published:** 2013-06-24

**Authors:** Sabrina Burkhardt, Linoj Kumar, Richard Chandra, Jack Saddler

**Affiliations:** 1 Forest Products Biotechnology/Bioenergy, 2424 Main Mall University of British Columbia, Greater Vancouver, Canada

**Keywords:** Forest residues, Chemical composition, Material balance, Bark, Extractives, Lignin, Steam pretreatment

## Abstract

**Background:**

Forest residues represent an abundant and sustainable source of biomass which could be used as a biorefinery feedstock. Due to the heterogeneity of forest residues, such as hog fuel and bark, one of the expected challenges is to obtain an accurate material balance of these feedstocks. Current compositional analytical methods have been standardised for more homogenous feedstocks such as white wood and agricultural residues. The described work assessed the accuracy of existing and modified methods on a variety of forest residues both before and after a typical pretreatment process.

**Results:**

When “traditional” pulp and paper methods were used, the total amount of material that could be quantified in each of the six softwood-derived residues ranged from 88% to 96%. It was apparent that the extractives present in the substrate were most influential in limiting the accuracy of a more representative material balance. This was particularly evident when trying to determine the lignin content, due to the incomplete removal of the extractives, even after a two stage water-ethanol extraction. Residual extractives likely precipitated with the acid insoluble lignin during analysis, contributing to an overestimation of the lignin content. Despite the minor dissolution of hemicellulosic sugars, extraction with mild alkali removed most of the extractives from the bark and improved the raw material mass closure to 95% in comparison to the 88% value obtained after water-ethanol extraction. After pretreatment, the extent of extractive removal and their reaction/precipitation with lignin was heavily dependent on the pretreatment conditions used. The selective removal of extractives and their quantification after a pretreatment proved to be even more challenging. Regardless of the amount of extractives that were originally present, the analytical methods could be refined to provide reproducible quantification of the carbohydrates present in both the starting material and after pretreatment.

**Conclusion:**

Despite the challenges resulting from the heterogeneity of the initial biomass substrates a reasonable summative mass closure could be obtained before and after steam pretreatment. However, method revision and optimisation was required, particularly the effective removal of extractives, to ensure that representative and reproducible values for the major lignin and carbohydrate components.

## Introduction

Various national and global incentives have been used to try and reduce our dependency on fossil derived transportation fuels while encouraging the production and use of renewable biofuels such as ethanol [1,2]. While virtually all of the ethanol currently used in automobiles is derived from sugar or starch crops there has also been a considerable investment in biomass-to-ethanol processes. A typical biomass-to-ethanol process involves the three major steps of pretreatment and fractionation, enzymatic hydrolysis of the cellulosic fraction and fermentation of the derived sugars to ethanol. Although many factors contribute to the overall costs of producing biomass derived ethanol, the feedstock cost has been reported to be among the highest [3]. One way to try and reduce these costs is by making use of underutilised biomass materials such as the residues obtained at forestry and saw/pulp mill sites. In British Columbia, a region rich in softwood biomass, there is an estimated 11 million dry tons surplus softwood-derived residues available annually [4].

The traditional methods of determining the composition of forest and agricultural derived biomass materials have been historically established by the pulp and paper and agricultural industries. From a forest biomass perspective the Technical Association of the Pulp and Paper Industry (TAPPI) has helped develop and standardized many of the methods used to characterize and quantify woody biomass. However, the main focus of the TAPPI methods are primarily to aid pulp producers determine the selectivity of chemical pulping, (such as the extent of delignification), maximizing pulp yield and strength as well as determining pulp bleachability [5,6]. As a result, there is less emphasis on determining a closed material balance or quantifying individual biomass components. For example, rather than using the sulfuric acid hydrolysis method developed by Peter Johan Klason for lignin isolation and quantification [7,8], the most commonly employed method used to determine the lignin content and bleachability of pulps is the indirect permanganate oxidation which does not provide an exact gravimetric measurement of pulp lignin content [9,10]. Similarly, the primary goal of measuring the composition of agricultural residues by the Association of Analytical Communities (AOAC International), formerly the Association of Official Agricultural Chemists, was to assess potential forage digestibility and its influence on animal nutrition [11]. Thus, both of the traditional woody and agricultural based methods for quantifying biomass have tended to be semi-quantitative as they were primarily used to determine those particular biomass characteristics that related to the final use of the starting substrate. The detailed tracking of the total starting material and each biomass component through a multi-step process was not a major focus of either the forest or agricultural based sectors until the oil crisis of the late 1970’s precipitated interest in the potential of producing fuels and chemicals from biomass. The National Renewable Energy Laboratory (NREL) in Golden, Colorado, has developed a comprehensive set of laboratory analytical procedures for characterising and quantifying biomass and these methods have been cited extensively in the bioconversion literature [12,13]. The main NREL recommended method for determining an accurate material balance involved modifying the established Klason procedure that uses a 72% sulfuric acid solution for primary hydrolysis at room temperature, followed by dilution with water and a secondary high temperature hydrolysis [14]. During this two stage analytical procedure, polysaccharides are almost quantitatively hydrolysed to soluble monosaccharides, leaving behind most of the lignin as an “insoluble residue” that is washed, filtered and measured gravimetrically. The dissolved monosaccharides are measured using chromatography techniques and the corresponding carbohydrate polymers are back calculated [14]. This simple procedure works best on “clean” biomass samples such as so-called white wood found in lumber or pulp chips, where mostly carbohydrates and lignin are present. However, as many lignocellulosic materials also contain components such as inorganics (ash), proteins and extractives, an appropriate set of extraction methods are typically used to remove and quantify these materials while minimising their interference with the acid hydrolysis step [15-17]. Despite some reported limitations with the recommended methods, the NREL Laboratory Analytical Procedures (LAP) provide a comprehensive set of protocols which can quantify the majority of the constituents present in a “typical” cellulosic biomass, while achieving a good summative mass closure with maximum ±5% variation reported between different labs [12,18].

However, compared to white wood, forest residues can contain significantly higher amounts of ash, extractives, lignin, and other ‘difficult-to-extract’ components such as suberin [19,20]. The amount, type and complexity of the extractives in forest residues (bark in particular) are substantially different from those of white wood and agricultural residues. The extractives content of white wood is generally lower and mostly comprised of lipophilic, fats and waxes, resins and terpenoids/steroids [21]. Due to their heterogeneity and inherent complexity, the composition of extractives is largely defined by the solvent used for extraction such as ethanol-benzene, acetone, ethanol or water. Thus it is difficult to use one extraction protocol to completely remove all of the polar and non-polar extractive components, especially in an extractive-rich biomass such as bark. In addition to the extractives, certain minor components such as acetyl groups, uronic acids, pectins and proteins can all play an important role in helping close the material balance. In previous studies where more heterogeneous feedstock’s were used, [22-24], the authors reported the difficulty in achieving a good material balance and in trying to quantify the individual components present in both the original biomass sample and during the pretreatment, fractionation and processing of the residues.

In the work described here we collected or prepared six different softwood residues (hog fuel I & II, logging residue (LR), interface fire slash (IFS), beetle-killed lodgepole pine wood chips (BK-LPP), and bark) and assessed how effective the NREL recommended methods [25] were in both providing a good material balance and quantifying some of the major biomass components. We also investigated how a “typical” pretreatment such as steam pretreatment might influence the robustness of the refined methods to provide a reasonable material balance including the reproducibility and accuracy of the mass closure and the recovery of the various biomass components. Different extraction strategies were also evaluated to see if they could enhance the accuracy of established methods when a more heterogeneous feedstock such as bark or hog fuel was used as the biomass feedstock.

## Results and discussion

### Physical characterisation and preparation of the forest derived residues

The residues were predominantly derived from Pacific Northwest softwoods species such as Douglas-fir, Western Hemlock and Lodgepole Pine. The Interface fire slash material was the only sample which had some hardwood residues and pine cones. The logging residue contained visible amounts of needles, some of which appeared partially decayed. As is described in Table 1, the residues also varied in their moisture content and particle size. To ensure a reproducible comparison the residues were first homogenised by milling to a similar particle size (2 mm) and conditioned to a uniform moisture content by soaking the material in water and subsequent vacuum filtration to remove the excess water (47-51% moisture content).

**Table 1:** The softwood-derived forest residues used in the work and their physical characteristics

	Source*	Moisture (% wt/wt) **	**Average size(mm×mm×mm)**	**Notes**
Lodgepole pine white wood (BKLPP)	Beetle-killed	7	25×25×5	Disturbance wood. Overall expected to be similar to white wood [26]
Interface fire slash (IFS)	Williams Lake, some aspen, mostly Douglas-fir and Pine	28	85×50×15	Juvenile wood contains thinner cell walls, shorter fiber length and higher lignin content [27,28]
Logging Residues (LR)	Williams Lake, mostly Lodgepole pine	42	80×25×10	Contained branches with higher ratio of compression wood. This will likely contribute to higher lignin content when compared to white wood. Will likely have more collapsed cell walls [28,29]
Hog fuel I (HOG I)	Olympic peninsula debarking debris, mostly Western Hemlock	62	40×5×2	Appeared to have a higher bark content. Expected to be challenging to process due to contamination.
Hog fuel II (HOG II)	Olympic peninsula debarking debris, woody urban waste, Western Hemlock	58	55×10×5	Primarily woody urban waste, which is extremely variable and may have higher ash content [30]
BARK	Lodgepole pine, freshly debarked	33	150×30×2	Reported to be high in extractives, high in lignin, low in carbohydrates, and higher in ash compared to white wood [19,23]

*All of these materials are mostly softwood derived.

**Microbial growth might occur in forest residues during storage with resulting sugar and extractive losses [31].

### Compositional analysis of the raw material

Initially, each of the six residue samples were analysed using the NREL LAP recommended compositional analysis method [12,18] without prior extraction. It was apparent that the total carbohydrates, lignin and ash together contributed 89 – 97% of the total dry weight of the starting materials depending on the source of the biomass (data not shown). Residues such as bark, which were anticipated to have a higher extractive content, gave the poorest mass closure.

This initial “Klason based method” was followed by the full NREL LAP method where the extractives were first quantified by a standard “water followed by ethanol” extraction prior to acid hydrolysis. This type of pre-extraction procedure is typically used to determine the extractive content of agricultural feedstocks [32,33] and the extractives in forest residues such as bark, which are known to be predominantly polar in nature [19,34]. When the extractive values were combined with the carbohydrate, lignin and ash values, an improved summative mass closure of 97 – 109% was obtained (Table 2). However, it was apparent that, some of the values were significantly higher than 100%. As the extractive values were determined from a separate analysis, the “double counting” of extractives from both the “overestimated lignin” values and extractive values themselves likely resulted in the observed, slightly higher summative mass closure. Although the reported summative values appeared to be close to 100%, it was likely that the mass closure was slightly overestimated due to the precipitation of the extractives with the lignin. Earlier work has shown that lignin is likely to be overestimated when extractives were present in the material quantified by Klason analysis [10,35]. The beetle killed lodgepole pine sample most closely resembles a typical “white wood” (Table 1) with the lower extractive content of this material minimising any interference with the lignin determination. In contrast, the highest value for mass closure was obtained with the bark sample, which contained the largest amount of extractives and therefore had the greatest probability of extractive precipitation with lignin during lignin quantification (Table 2). Previous work has also shown that, in addition to overestimating the amount of lignin present, extractives and ash can also influence the carbohydrate analyses [15]. However, all of the forest derived residues contained little ash (less than 7% ash) and little or no influence was anticipated.

**Table 2:** **Chemical composition of the raw materials before steam pretreatment** (% **dry weight) (Carbohydrates and lignin analysis were completed prior to extraction)**

	**Arabinan**	**Galactan**	**Glucan**	**Xylan**	**Mannan**	**Lignin**		Extractives***	Ash****	**Sum**
						**Acid insoluble**	**Acid soluble**			
BKLPP*	1.6 (0.2)**	2.7 (0.2)	42.4 (0.3)	5.9 (0.6)	11.4 (0.1)	28.2 (0.8)	0.4 (0.0)	3.8 (0.2)	0.1 (0.0)	97.4 (1.1)
IFS	1.9 (0.0)	5.6 (0.1)	36.1 (0.8)	6.6 (0.1)	9.9 (0.2)	33.3 (0.4)	0.6 (0.0)	6.3 (1.1)	0.1 (0.0)	99.1 (1.5)
LR	2.4 (0.3)	3.0 (0.1)	33.6 (1.0)	5.5 (0.2)	7.8 (0.2)	38.6 (0.7)	1.1 (0.1)	9.4 (0.0)	0.4 (0.2)	102.3 (1.4)
HOG I	1.8 (0.1)	1.8 (0.1)	29.6 (0.3)	4.5 (0.0)	6.0 (0.2)	42.8 (0.1)	1.2 (.1)	6.2 (0.2)	6.9 (0.0)	100.7 (0.5)
HOG II	1.2 (0.0)	1.9 (0.0)	37.5 (0.7)	4.6 (0.1)	7.9 (0.2)	39.7 (0.2)	1.0 (0.1)	5.7 (0.3)	2.9 (0.1)	102.1 (0.8)
BARK	6.4 (0.0)	3.5 (0.0)	19.2 (0.0)	3.1 (0.0)	1.5 (0.0)	52.7 (0.5)	1.2 (0.0)	19.0 (1.5)	2.0 (0.2)	109.2 (1.6)

*BKLPP refers to Beetle-killed lodge pole pine white wood; IFS is Interface Fire Slash (or Forest Thinnings); LR refers to Logging Residue.

HOG I & II are two different types of hog fuels collected from Nippon Paper. Hog I is more bark intensive. Bark samples are sourced from lodge pole pine wood.

**Values in the bracket represent the standard deviations of triplicate analysis.

***Extractive values reported are from a separate analysis and the quantification of other biomass components were carried out on the raw material without any prior extraction.

****Ash values reported are from biomass samples prior to extraction.

To try to better determine the extent of extractive interference in achieving an accurate material balance in each of the residue samples, a two stage water-ethanol extraction process was next assessed. As was anticipated, prior removal of the extractives had a substantial effect on the compositional analysis of the forest residues and the determined lignin content decreased significantly (3 – 18%) (Tables 3 and 4). As was also expected, the interference due to extractives was considerably higher for the bark and logging residues as these substrates contained greater amounts of extractives.

**Table 3:** **Chemical composition of the raw materials** (% **dry weight of the original biomass**)*

	**Arabinan**	**Galactan**	**Glucan**	**Xylan**	**Mannan**	**Lignin**		**Extractives**	Ash****	**Sum**
						**Acid insoluble**	**Acid soluble**			
BKLPP**	1.3 (0.0)***	2.2 (0.0)	42.3 (0.7)	5.5 (0.1)	13.0 (0.2)	24.9 (1.2)	0.2 (0.0)	3.8 (0.2)	0.1 (0.0)	93.2 (1.4)
IFS	1.4 (0.0)	2.3 (0.0)	37.6 (1.0)	6.3 (0.2)	10.3 (0.3)	23.5 (0.7)	0.2 (0.0)	6.3 (1.1)	0.1 (0.0)	88.2 (1.7)
LR	1.5 (0.0)	2.7 (0.0)	35.5 (0.9)	5.2 (0.1)	10.8 (0.3)	25.8 (1.2)	0.3 (0.1)	9.4 (0.0)	0.4 (0.2)	91.7 (1.5)
HOG I	1.0 (0.0)	1.8 (0.0)	31.5 (0.2)	4.5 (0.0)	7.1 (0.1)	36.1 (0.2)	0.6 (0.1)	6.2 (0.2)	6.9 (0.0)	95.9 (0.4)
HOG II	0.8 (0.0)	1.8 (0.1)	36.7 (1.6)	4.5 (0.3)	9.0 (0.5)	33.8 (0.2)	0.5 (0.0)	5.7 (0.3)	2.9 (0.1)	96.2 (1.5)
BARK	4.7 (0.1)	3.0 (0.0)	19.0 (0.3)	3.1 (0.0)	1.7 (0.1)	34.5 (0.7)	0.7 (0.0)	19.0 (1.5)	2.0 (0.2)	87.5 (1.7)

*Carbohydrates and lignin analysis completed after successive extractions with water and ethanol.

**BKLPP refers to Beetle-killed lodge pole pine white wood; IFS is Interface Fire Slash (or Forest Thinnings); LR refers to Logging Residue.

HOG I & II are two different types of hog fuels collected from Nippon Paper. Hog I is more bark intensive. Bark samples are sourced from lodge pole pine wood.

***Values in the bracket represent the deviations of triplicate analysis.

****Ash values reported are from biomass samples prior to extraction.

**Table 4:** **The influence of an extraction step on the lignin and carbohydrate content of the original biomass (% dry weight of the original biomass**)

	**Extractives**		**Acid insoluble lignin**			Hemicellulose**		**Glucan**	
**Extraction**	**Water**	**Water + Ethanol**	**Unextracted**	**After water extraction**	**After water followed by ethanol extraction**	**Unextracted**	**After water followed by ethanol extraction**	**Unextracted**	**After water followed by ethanol extraction**
BKLPP	2.6 (0.2)*	3.8 (0.2)	28.2 (0.8)	25.0 (0.8)	24.9 (1.2)	21.6 (0.6)	22.0 (0.2)	42.4 (0.3)	42.3 (0.7)
IFS	4.1 (0.3)	6.3 (1.1)	33.3 (0.4)	26.7 (0.3)	23.5 (0.7)	24.0 (0.2)	20.3 0.4)	36.1 (0.8)	37.6 (1.0)
LR	5.2 (0.3)	9.4 (0.1)	38.6 (0.7)	28.2 (0.1)	25.8 (1.2)	18.7 (0.4)	20.2 (0.3)	33.6 (1.0)	35.5 (0.9)
HOG I	3.6 (0.1)	6.2 (0.2)	42.8 (0.1)	39.8 (0.5)	36.1 (0.2)	14.1 (0.3)	14.4 (0.1)	29.6 (0.3)	31.5 (0.2)
HOG II	3.5 (0.7)	5.7 (0.3)	39.7 (0.2)	35.4 (1.8)	33.8 (0.2)	15.6 (0.2)	16.1 (0.6)	37.5 (0.7)	36.7 (1.6)
BARK	13.6 (1.2)	19.0 (1.5)	52.7 (0.5)	39.4 (1.1)	34.5 (0.7)	14.5 (0.1)	12.5 (0.1)	19.2 (0.0)	19.0 (0.3)

*Numbers in the bracket represent standard deviations of triplicate analysis.

**Hemicellulose represents the sum of arabinan, galactan, xylan and mannan.

The data indicated that prior water-ethanol extraction resulted in a summative mass closure of 88 – 96% and that the lowest sum was observed with bark, forest thinning’s and logging residues, likely due to some missing components which were not accounted for in the analysis (Table 3). One of the components that was likely not picked up in these three samples is pectin which would be detected as uronic acid [18,36]. However, the uronic acid content was not analyzed in this study. As mentioned previously, the interface fire slash contained a blend of juvenile wood samples from both softwoods and hardwoods (Table 1). Hardwood hemicellulose is generally more acetylated and therefore should have some acetyl groups, which were also not quantified. The bark sample gave the lowest mass closure, possibly be due to residual extractives which were still present in the substrate even after a water-ethanol extraction step (Table 3). These residual extractives may have been solubilized in the concentrated acid and thus not accounted for during a normal Klason analysis.

It was apparent that trying to obtain a good material balance of high extractive containing forest biomass sample without a prior extraction step resulted in an overestimation of the lignin but only minor variations in the carbohydrate content (Table 4). The largest variation occurred in determining the hemicellulose content of the bark and IFS samples, where a respective 2 and 4% loss seem to have resulted from the two stage extraction (Table 4). This loss was likely due to the solubilisation of the neutral sugars present in the pectin component of these materials, as they can be relatively easily removed by hot water hydrolysis [15,19].

The water-ethanol procedure recommended in the NREL LAP method was primarily developed with agricultural residues in mind [32]. In contrast, probable forest residue feedstocks such as bark or hog fuel, are known to contain extractives which cannot be entirely solubilized by a simple water-ethanol extraction [37]. Even for agricultural and whitewood feedstocks, large variations in extractive content have been reported between different laboratories [18]. For the forest residues studied here it is possible that, even after a water-ethanol extraction, residual extractives might be hydrolysed in the concentrated acid medium or may precipitate and interfere with lignin quantification. It has also been shown that significant amounts of both polar and non-polar extractable components are present in these types of biomass [5,8,19,38]. For example poly flavonoids, terpenes, resin acids, fats, and suberin are all found in bark due to the protective, anti-fungal/insect properties they provide the tree. Due to the diversity and quantity of extractives in bark, a broad spectrum of methods have been developed to remove and characterize the different types of extractives [20,34,39,40]. Mild alkali has been reported to be one of the most effective methods for the removal of most of the extractives with minimal influence on subsequent assessment of the carbohydrate content [41,42]. The partial depolymerisation and the increased ionization of the high molecular weight extractive components (such as polyphenols) increase their solubility during alkaline extraction [41,42]. A 1.0% NaOH solution in reflux has often been used for maximum extractive removal from bark and to provide a more realistic estimation of the lignin content [43]. Therefore, we next applied an alkali extraction to the untreated bark and hog fuel to determine if this approach might enhance the summative mass closure.

The bark and hog fuel samples were shown to contain 43 and 24% alkali-soluble extractives respectively (Table 5) with the bark values similar to those found previously with pine bark [34,40]. The alkali extraction further reduced the lignin content of the original material to 21 and 28% respectively for the bark and hog fuel samples, a 14% and 8% further reduction in lignin content when compared to the lignin values determined after water-ethanol extraction. This seemed to indicate that alkali extraction effectively solubilised most of the extractives and resulted in a much better summative mass closure of 96 and 98% respectively for both the bark and hog fuel substrates. It was also likely that the hydrolysis of extractive components such as suberin and long chain fatty acids, and their subsequent dissolution in the alkaline solution, was representative of the efficiency of removal of the majority of the extractive compounds [42]. However, the alkali extraction did result in the loss of some of the hemicellulosic sugars, particularly arabinose and galactose (Table 5). About 3 and 2% arabinan and galactan appeared to have been extracted in alkali, which could be attributed to the more efficient extraction of pectins in an alkaline medium [44]. Overall, alkali extraction resulted in a significantly better summative mass closure for most of forest residues particularly the bark sample.

**Table 5:** **Chemical composition of bark and hog fuel based on alkali extraction prior to compositional analysis(% dry weight of the original biomass**)*

	**HOG I**	**BARK**
Arabinan	1.2 (0.0)**	3.6 (0.0)
Galactan	1.5 (0.0)	2.1 (0.0)
Glucan	29.4 (0.2)	19.2 (0.3)
Xylan	4.1 (0.1)	3.2 (0.0)
Mannan	4.5 (0.2)	1.5 (0.0)
Acid insoluble lignin	27.6 (0.7)	20.1 (0.7)
Acid soluble lignin	0.3 (0.0)	0.3 (0.0)
Ash***	6.9 (0.0)	2.0 (0.2)
Extractives	23.5 (0.7)	42.9 (0.9)
Mass closure	99.4 (1.2)	95.8 (1.0)

*Carbohydrates and lignin analysis completed after extraction with 1% NaOH and subsequently the values are expressed as g per 100 g of original material.

**Values in the bracket represent the deviations of three replicate analysis.

***Ash values reported are from biomass samples prior to extraction.

### Influence of steam pretreatment on determining the chemical composition of the forest residue samples

As previous work had shown that pretreatment could influence the ability to achieve a good mass closure due to factors such as degradation reactions producing materials such as pseudolignins [35], we next assessed whether steam pretreatment of the forest residue substrates might influence the robustness of the compositional analysis and our ability to achieve reasonable mass balance closure. The substrates were subjected to two different steam pretreatment conditions (low and high severity, 180°C and 200°C) for 5 minutes with 4% SO_2_ impregnation levels. After steam pretreatment, the water insoluble fraction was subjected to a chemical compositional analysis (Tables 6 and 7). However, unlike the starting material, the water insoluble, cellulosic rich component cannot be subjected to an extraction procedure. Depending on the severity of the applied conditions, steam pretreatment typically results in significant depolymerisation of the lignin component, leading to a substantial reduction in its molecular weight. A subsequent extraction carried out after steam pretreatment will likely solubilise a significant fraction of this depolymerised lignin, making it extremely difficult to selectively remove just the extractive components [45,46]. Although the compositional analysis of the water insoluble component was carried out without any prior extraction, the summative mass closure obtained was reasonably good ranging from 96 – 101% for the low severity (Table 6) and 89 – 100% for the high severity conditions (Table 7).

**Table 6:** **Chemical composition of the water insoluble component after the steam pretreatment at 180**°**C**, **5 minutes 4**% **SO**_**2 **_**(% dry weight of water insoluble solids**)

	**Arabinan**	**Galactan**	**Glucan**	**Xylan**	**Mannan**	**Lignin**	**Ash**	**Sum**
BKLPP*	0.4 (0.1)**	0.8 (0.1)	49.6 (0.1)	3.5 (0.2)	4.6 (1.0)	38.2 (0.9)	0.1 (0.1)	97.2 (0.6)
IFS	0.8 (0.0)	1.5 (0.0)	44.5 (0.4)	5.2 (0.0)	5.3 (0.0)	38.2 (0.2)	0.5 (0.3)	96.0 (1.4)
LR	0.7 (0.0)	1.3 (0.0)	43.6 (0.5)	3.4 (0.0)	4.3 (0.1)	41.7 (0.7)	0.6 (0.0)	95.6 (0.9)
HOG I	0.3 (0.0)	0.8 (0.0)	36.8 (0.5)	3.2 (0.0)	3.6 (0.0)	51.0 (0.8)	3.8 (0.2)	99.5 (0.8)
HOG II	0.2 (0.0)	1.0 (0.0)	42.4 (0.4)	3.8 (0.1)	5.9 (0.1)	45.1 (0.3)	2.5 (0.7)	100.9 (1.9)
BARK	3.6 (0.0)	2.4 (0.0)	22.2 (0.5)	3.2 (0.0)	1.4 (0.0)	64.6 (1.8)	2.0 (0.4)	99.4 (0.8)

*The raw materials were ground to a particle size of ~2 mm and moisture content was adjusted to ~50% by wet weight of the sample prior to pretreatment.

**Numbers in the bracket represent standard deviations of triplicate analysis.

**Table 7:** **Chemical composition of the water insoluble component after the steam pretreatment at 200**°**C**, **5 minutes 4**% **SO**_**2 **_**(% dry weight of water insoluble solids**)

	**Arabinan**	**Galactan**	**Glucan**	**Xylan**	**Mannan**	**Lignin**	**Ash**	**Sum**
BKLPP*	0.3 (0.0)**	0.4 (0.1)	51.7 (1.0)	1.0 (0.1)	1.7 (0.2)	39.8 (0.5)	0.3 (0.1)	95.2 (0.8)
IFS	0.4 (0.0)	0.7 (0.0)	45.2 (0.3)	2.3 (0.0)	2.3 (0.1)	39.8 (1.8)	0.5 (0.7)	93.9 (0.3)
LR	0.3 (0.0)	0.7 (0.0)	42.0 (0.5)	1.2 (0.0)	1.8 (0.0)	45.4 (1.4)	0.6 (0.2)	95.4 (0.5)
HOG I	0.3 (0.0)	0.6 (0.1)	31.1 (0.9)	1.9 (0.1)	2.1 (0.1)	50.9 (1.1)	5.0 (0.0)	100.1 (0.9)
HOG II	0.1 (0.0)	0.4 (0.0)	42.7 (0.5)	1.7 (0.1)	2.6 (0.1)	42.5 (1.3)	1.5 (0.1)	99.7 (0.5)
BARK	2.6 (0.2)	1.4 (0.1)	22.4 (0.8)	1.4 (0.1)	0.9 (0.1)	58.5 (1.6)	1.9 (0.4)	89.1 (0.1)

*The raw materials were ground to a particle size of ~2 mm and moisture content was adjusted to ~50% by wet weight of the sample prior to pretreatment.

**Numbers in the bracket represent standard deviations of triplicate analysis.

**Figure 1:**
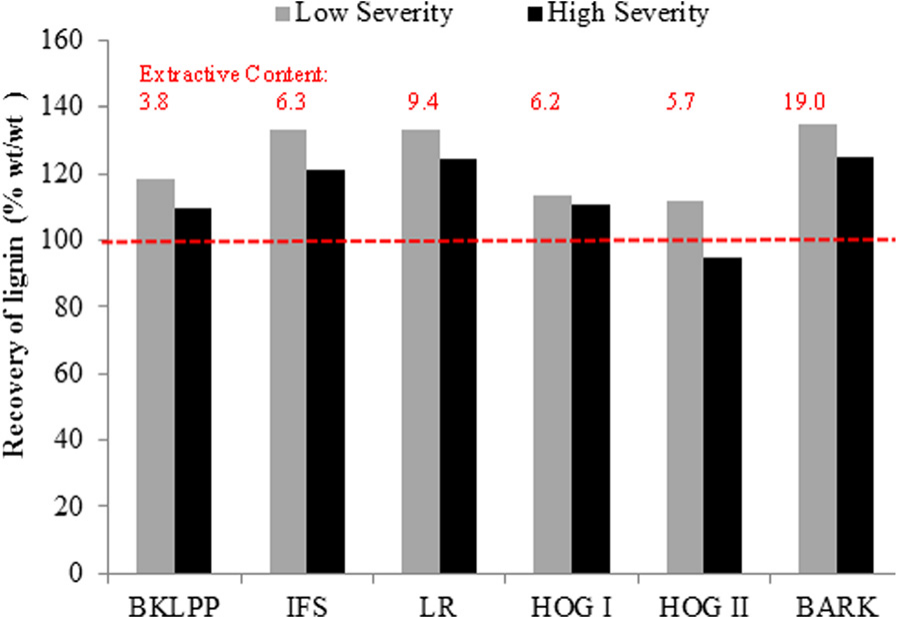
**Influence of extractives on the recovery of lignin in the water insoluble component after the steam pretreatment at 180 and 200°C.**

The reasonable mass balance closure obtained (Tables 6 and 7) implied that most of the extractives were volatilised or solubilised during steam pretreatment and any remaining extractives were quantitatively precipitated with the lignin during analysis. The solids recovery obtained after steam pretreatment ranged from 65-85%, similar to the recoveries previously reported with other softwood feedstocks [26,47]. In general, the amount of lignin that was detected in the water insoluble component after steam pretreatment was slightly higher than that measured in the original material (Figure 1). This was likely due to the precipitation of extractives with the lignin during steam pretreatment leading to higher lignin values being measured. Earlier work had shown that some of the extractives condense with the lignin during steam pretreatment as well as during a subsequent Klason analysis, thus increasing the reported lignin values [35]. However, it is likely that the solubility of the extractives and their precipitation with lignin will be influenced by the severity of steam pretreatment conditions used. It appears that pretreatment at 180°C did not sufficiently fragment and solubilise the extractives, leaving most of them in their native form and allowing their precipitation with the lignin during Klason analysis. The more severe steam pretreatment at higher temperatures likely depolymerised the extractives, resulting in their dissolution and reduced presence in the insoluble biomass and thus not contributing to the lignin quantification.

### Sugar recovery during steam pretreatment and the suitability of the materials for bioconversion

**Figure 2:**
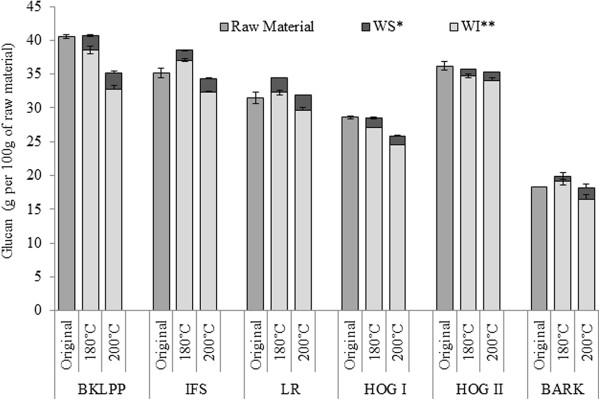
**Recovery of original glucan after the steam pretreatment at two different severities (200°C, 5 minutes and 4% SO**_**2; **_**180°C, 5 minutes and 4% SO**_**2**_**).** *Water soluble component after the pretreatment. ** Water insoluble cellulosic component after the steam pretreatment. The glucose present in the water soluble components of 180°C and 200°C pretreatments had 80-90% and 0-45% oligomeric sugars respectively. The error bars represent the standard deviations of triplicate analysis.

**Figure 3:**
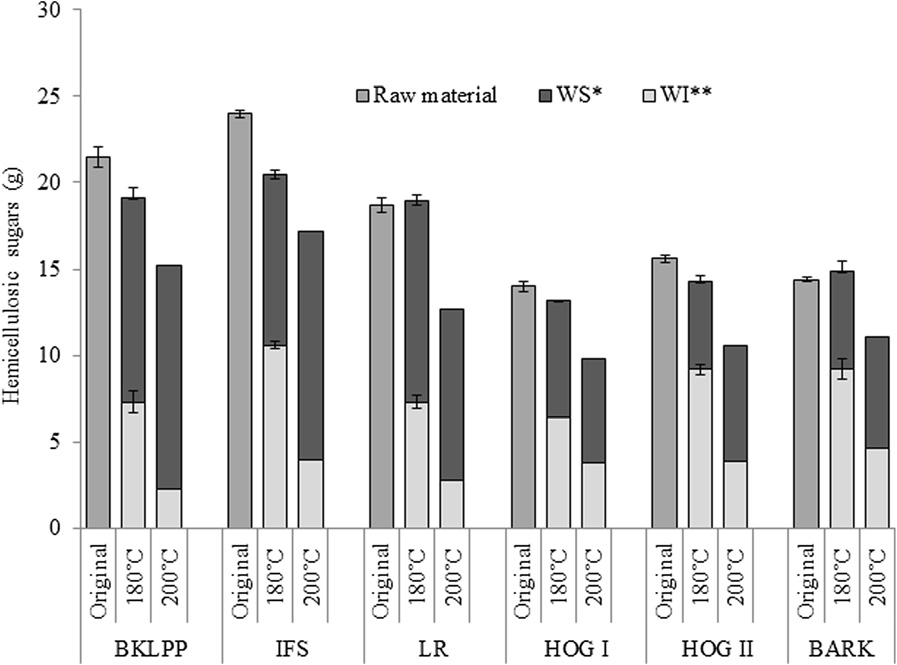
**Recovery of original hemicellulosic sugars after the steam pretreatment at two different severities (200°C, 5 minutes and 4% SO**_**2; **_**180°C, 5 minutes and 4% SO**_**2**_**).** *Water soluble component after the pretreatment. ** Water insoluble cellulosic component after the steam pretreatment. Hemicellulose represents the sum of arabinan, galactan, xylan and mannan. The sugars present in the water soluble components of 180°C and 200°C pretreatments had 53-80% and 5-45% oligomeric sugars respectively. The error bars represent the standard deviations of triplicate analysis.

Unlike the problems encountered with the lignin quantification, good reproducibility and mass balance was generally obtained with the carbohydrate values. Both of the pretreatment severities that were assessed resulted in near complete glucan recovery (>90%) in the combined water soluble and insoluble fractions (Figure 2). The total hemicellulose recovery at the lower severity was 85-100%, while the recovery decreased to 68-77% after treatment at the higher severity, although most of the hemicellulose was recovered in a monomeric form (>55%) after treatment at the higher severity (Figure 3). This should allow their ready fermentation without the need to further hydrolyse the oligomeric sugars while the solubilization of most of the hemicellulose should enhance the accessibility of the cellulase enzymes to the cellulose [48,49].

When evaluating the potential of forest residues as candidate substrates for a biomass-to-ethanol process the theoretical sugar/ethanol yield that can be anticipated will likely be significantly lower than what could be expected using white wood. However, with the exception of the bark sample, which had a significantly lower carbohydrate content, all of the other residues still contained 43-64% polysaccharides. In addition to a lower theoretical sugar/ethanol yields, the enzymes and yeast used in the conversion can be significantly inhibited by the extractives and lignin in bark [23,50]. However, extractives such as tannins, have potential higher value applications such as phenolic resins and pharmaceutical/nutraceuticals uses [37,42,51] and lignin can be used to make phenolic and epoxy resins, carbon fibers and several other valuable products [20,52]. Therefore the selective fractionation and removal of extractives and lignin might not only aid in achieving a better material balance for pretreated forest residues it might also help us derive higher-value co-products while providing a “carbohydrate enriched” fraction that could be used as the sugar feedstock for fuel and chemical.

## Conclusion

Despite the challenges resulting from the heterogeneity of the six different forest residues, a reasonable summative mass closure could be obtained before and after steam pretreatment. However, method revision and optimisation was required, particularly for the effective removal of extractives from the raw material to ensure that representative and reproducible values for the major lignin and carbohydrate components could be derived. With the increasing realisation that the extractive components of biomass are in themselves potentially valuable chemical feedstock’s, further improvements in the solvents and extractive procedures used to characterize the various extractives should help achieve both improved mass balance closure and better utilisation of the individual components of the extractives fraction.

## Methods

### Description of the forest residues

Six different forest derived residues were collected including; two hog fuels Hog I and Hog II (from Nippon Paper, Port Angeles, Washington St., USA, sampled from two different batches and varied in their Western Hemlock debarking debris and woody urban waste (delivered from Rainier Urban and Hermann Local) content; logging Residue (LR, chipped onsite and collected by Pioneer Biomass from 100km east of Williams Lake, BC); Forest Thinnings (FT) also known as Interface Fire Slash (IFS) (chipped fresh onsite at Williams Lake and consist of primarily Douglas fir and Pine, with some Aspen); Beetle-killed lodgepole pine (BK-LPP) white wood chips (from Tolko Industries Ltd Vernon, BC (average tree age 101 ± 20 years)); Lodgepole pine bark obtained by debarking freshly cut BK-LPP logs in the UBC process development unit. The moisture content of the biomass samples (as received) varied from 7–60%. All samples were frozen upon arrival to reduce the effect of potential degradation due to storage. To ensure homogeneity within the residues for steam pretreatment, the samples were air dried before grinding two a 2 mm diameter by Wiley mill and then rewet to “green wood” moisture (50%) prior to use.

### Pretreatment

Prior to steam pretreatment, the ground samples were impregnated by adding a specified amount of SO_2_ (4% wt/wt of the substrate [26]) to sealable plastic bags containing 150 dry grams of the biomass. Once impregnated, the bags were immediately sealed and left for 1 h before opening and venting under the fume hood for half an hour to displace any unabsorbed SO_2_ prior to steam pretreatment. Steam pretreatment was conducted in a 2 L StakeTech steam gun at 200 and 180°C for 5 min. After the pretreatment, the whole slurry was removed and the water soluble and insoluble fractions were separated by vacuum filtration. The water insoluble fraction was thoroughly water washed and the water washed solids were subsequently vacuum filtered. The final moisture content of the water insoluble fraction was within the range of 60-80%.

### Analytical methods

For all compositional analyses, the NREL LAP method [53] for sample preparation was followed in accordance to methods used for softwood feedstocks, unless otherwise specified.

For the raw material compositional analysis, water and ethanol soluble extractives were quantified using NREL’s LAP [32] with the following clarifications. Ten grams of oven-dried, 40-mesh ground biomass samples were extracted for 24 h with water at approximately 6 cycles/h. The water in the round-bottomed flask was then dried in the oven at 105°C for 24 h to determine the weight of extractives present in the sample flasks. The same biomass was also dried in the 105°C oven before being subjected to ethanol extraction by the same method. The ethanol extract was first evaporated to dryness in fumehood, at room temperature, and subsequently placed in the oven overnight to ensure complete removal of the residual moisture/solvent from the material. The extractives are subsequently weighed to determine the amount of ethanol soluble extractives. Alkali extraction was completed in a 1:20 ratio of solid : liquid, with 5 grams in 1% NaOH in water at reflux for 2 hours [42].

Ash analysis was completed in a muffle furnace at 550°C for 5 h to determine inorganic solids. This followed the NREL LAP method [53] and was completed both prior to and after completing water and ethanol extractions in the untreated biomass samples.

Moisture contents were determined by drying to a constant weight at 105°C in a convection oven. The Klason lignin content and the structural carbohydrates present in the pretreated solid (water insoluble fraction) and raw material substrates were determined according to the NREL LAP method [14]. When analysing the chemical composition of the pretreated materials, a second chemical compositional analysis of the raw/untreated material were also run in parallel in order to make a direct comparison and obtain a more accurate material balance. The acid-soluble lignin was determined by UV absorption at 205 nm as also described by NREL [14]. The monosaccharide content was determined using a DX-3000 high-performance liquid chromatography (HPLC) system (Dionex, Sunnyvale, CA), equipped with an anion exchange column (Dionex CarboPac PA1), and using fucose as the internal standard. The column was eluted with deionized water at a flow rate of 1 ml/min. Aliquots (20 μl) were injected after being passed through a 0.45-μmnylon syringe filter (Chromatographic Specialties Inc., Brockville, ON, Canada). The baseline stability and detector sensitivity were optimized by post column addition of 0.2 M NaOH at a flow rate of 0.5 ml/min using a Dionex AXP pump. The column was reconditioned using 1 M NaOH after each analysis. The monosaccharides in the substrates were quantified with reference to standards. The sugar standards were autoclaved in parallel with samples to correct for possible decomposition during Klason lignin and carbohydrate determination. All analyses were completed in triplicate.

The proportion of oligomeric sugars present in the water soluble fraction was analyzed by subjecting the liquid to a 4% sulfuric acid hydrolysis in an autoclave [54]. As explained in Klason analysis procedure, standards were run in parallel to correct for any hydrolysis loss factors. The monomeric sugars present in the sample, as measured by HPLC, were subtracted from the total sugars to obtain the oligomer content of the liquid.

## Abbreviations

AOAC: Association of Official Agricultural Chemists; BK-LPP: Beetle-killed lodgepole pine wood chips; HOG: Hog fuel; IFS: Interface fire slash; HPLC: High pressure liquid chromatography; LR: Logging residue; NREL-LAP: National renewable energy lab’s laboratory analytical procedures; TAPPI: Technical association of the pulp and paper industry.

## Competing interests

The authors declare that they have no competing interests.

## Authors’ contributions

All of the authors contributed equally to most aspects of the work reported in the manuscript. SB carried out most of the laboratory work with guidance from LK and RC. The research was conceptualised and planned by LK and JS, with these authors and SB contributing to interpretation of results and drafting of the paper. All authors read and approved the final manuscript.
